# Antioxidant and Anti-Inflammatory Agents from the Sea: A Molecular Treasure for New Potential Drugs

**DOI:** 10.3390/md20020132

**Published:** 2022-02-10

**Authors:** Marzia Vasarri, Donatella Degl’Innocenti

**Affiliations:** Department of Experimental and Clinical Biomedical Sciences, University of Florence, Viale Morgagni 50, 50134 Florence, Italy; donatella.deglinnocenti@unifi.it

Nowadays, natural compounds are widely used worldwide for the treatment of human diseases and health disorders. Throughout history, plants have been the primary sources of many pharmaceutical agents. However, over the years, great attention has been paid to the incredible biodiversity of life in seas, which has proven to be an exceptional reservoir of novel bioactive molecules with disparate structural and chemical characteristics, and a source of inspiration for new drug discovery.

To date, several marine drugs have been pharmacologically approved for the treatment of various diseases, while many other compounds are in clinical trials. At the same time, the global preclinical marine pharmaceutical pipeline involves research with more than 1000 marine chemicals with diverse biological properties.

The treasures of the sea have provided fundamental contributions to modern medicine, supplying important scientific discoveries for human health. Thus, in recent decades, marine bio-discovery has become “frontier” research for many scientists in academia and industry.

Today, it is well recognized that inflammation and oxidative stress are two closely related biological processes. The impact of inflammation and related oxidative stress is a huge issue in human health, as most chronic diseases and disorders are deeply linked to the interaction between these two biological phenomena.

In the relentless demand to discover new safe and effective agents with anti-inflammatory and antioxidant properties beneficial to human health, the marine environment has emerged as an unexplored molecular treasure trove.

The Special Issue “Marine Anti-Inflammatory and Antioxidants Agents 2021” collected the latest research, both in vitro and in vivo, on natural compounds from a variety of deep-sea organisms (including arthropods, oysters, mussels, algae, microalgae and cyanobacteria) with anti-inflammatory and/or antioxidant properties as potential candidates for new drug discovery, and in general for the field of marine biotechnology.

Among the natural occurring biomolecules, peptides are natural products present in many marine species. A large amount of scientific evidence reports that marine peptides have a high nutraceutical and medicinal power thanks to their wide spectrum of biological properties, as in the case of peptides from oyster hydrolysate (OPs). Oysters, the largest farmed shellfish in the world, are a good source of polypeptides, with antioxidant, immune, antimicrobial, antitumor, antifatigue and hepatoprotective properties. The work of Zhang et al. (2021) adds new insight to the benefits of peptides from oyster (*Crassostrea hongkongensis*) hydrolysate by describing their protective effect on testicular injury and disorders of spermatogenesis caused by tryptolide (TP) [1]. In mice with testicular injury, the ingestion of OPs for 4 weeks significantly improved the sperm count and motility of mice, and alleviated seminiferous tubule injury. OPs exerted antioxidant properties by upregulating the Nrf2 signaling pathway and thereby promoting the activity of antioxidant enzymes and the expression of antioxidant enzyme regulatory proteins in the testis. The activities of enzymes related to energy metabolism in the testis also improved after the ingestion of OPs, and serum hormone levels returned to normal. Severe testicular tissue damage improved due to the OP-induced inhibition of the JNK signaling pathway and Bcl-2/Bax-mediated apoptosis. For the first time, this study provides evidence on the potential improvement of male reproductive function by OPs, and discloses the experimental basis for the development of OPs in functional foods.

Siregar et al. (2021) also directed their research on the beneficial properties of OPs for human health [2]. Specifically, the authors explored the mechanism of hepatoprotective action of the peptide tyrosine-alanine (YA), identified as the main component of oyster (*Crassostrea gigas*) hydrolysate, in a mouse model with liver injury induced by intraperitoneal injection with lipopolysaccharide (LPS) and d-galactosamine (d-GalN). It has been demonstrated that the pre-administration of YA (50 mg/kg) significantly reduced inflammatory, apoptotic, ferroptotic and pyroptotic liver injury induced by the intraperitoneal injection of LPS/d-GalN showing hepatoprotective effects. This study provides clear evidence of YA as a potential hepatoprotective bioactive peptide in acute liver injury, such as acute or fulminant liver failure, and acute hepatitis.

The study of peptides as potential functional agents in human health was also undertaken by Suryaningtyas et al. (2021) [3]. Here, the authors demonstrated the cytoprotective activity of two peptides identified as FTVN and EPTF from the blue mussel (*Mytilus edulis*) and their role in preventing endothelial dysfunction (ED) mediated by oxidative stress induced by H_2_O_2_ exposure in human umbilical vein endothelial (HUVEC) cells. The investigation of the cytoprotective mechanism of these two peptides and their combination revealed that the peptides significantly reduced HUVEC death caused by H_2_O_2_ exposure through the enhancement of the antioxidant defense system via the upregulation of the cytoprotective enzyme heme oxygenase-1, and antinecrotic action. In light of these data, the authors suggest potential applications of peptides from the blue mussel as functional agents in protecting against ED-mediated oxidative stress.

Taken together, these briefly summarized works shed light on the possibility of using bioactive peptides and peptide-rich protein hydrolysates as an alternative to synthetic drugs for the prevention and treatment of acute and chronic diseases. However, further in vivo research will be required to develop a nutraceutical or pharmaceutical component based on these findings.

Among the marine-derived proteins studied over the years for their human health benefits, microalgae pigments (or phycobiliproteins) are well known. C-phycocyanin has been widely described as a phycobiliprotein with nephroprotective activity due to its antioxidant properties. Among others, C-phycoerythrin (C-PE), an oligomeric chromoprotein from cyanobacteria, is also already used in the food and cosmetic industries, as well as in diagnosis and research due to its nutraceutical properties, e.g., scavenging and antioxidant activity. Blas-Valdivia et al. (2021) contributed to the study of the biological properties of phycobiliproteins by revealing, for the first time, that the nephroprotective activity of C-PE (purified from *Phormidium persicinum*) is closely related to its antioxidant activity in the kidney of animal models of HgCl_2_-induced acute kidney injury [4]. Specifically, C-PE has been shown to act by preventing the HgCl_2_-induced increase in oxidative stress. In addition, C-PE prevents podocyte destruction and damage to glomerular and tubular cells by acting on intracellular signaling pathways involved in proteostasis.

As with terrestrial organisms, carotenoids represent the most common group of pigments in marine environments. Marine carotenoids exert strong antioxidant, restorative, antiproliferative and anti-inflammatory effects and can be used both as skin photoprotection to inhibit the negative effects of solar UV radiation and as nutraceutical/cosmeceutical ingredients to prevent oxidative stress-related diseases. In this regard, Yin et al. (2021) contributed with the publication of two research articles focused on the biological properties of astaxanthin, a naturally occurring red carotenoid pigment classified as xanthophyll native to marine organisms, such as microalgae and seafood, with known potent antioxidant properties [5,6]. The authors evaluated the anti-inflammatory and antioxidant capacity of astaxanthin on the immune functions of murine dendritic cells (DCs) in a sepsis model. Astaxanthin was shown to protect DCs from LPS-induced immune dysfunction. Specifically, it reduced the expression of LPS-induced inflammatory cytokines and phenotypic markers of DCs. It promoted endocytosis in LPS-treated DCs and hindered LPS-induced DC migration and abrogated allogeneic T-cell proliferation. Astaxanthin inhibited the LPS-induced immune dysfunction of DCs through the activation of the HO-1/Nrf2 axis, and when administered orally, it increased the survival rate of LPS-affected mice in vivo [5]. Furthermore, the authors demonstrated that astaxanthin, again via the HO-1/Nrf2 axis, protected LPS-induced DCs and LPS-affected mice from oxidative stress to achieve overloaded inflammatory control [6]. These studies lend strength to astaxanthin as a potential drug candidate for applications in various inflammatory diseases.

Historically, whole plants or mixtures of plants were used in traditional medicine rather than isolated compounds. There is evidence that crude extracts often have greater bioactivity in vitro and/or in vivo than isolated constituents at an equivalent dose. This effect could be due to the positive interactions between the components of whole extracts, as opposed to the activity of the individual compound. In this regard, Bigagli et al. (2021) performed a comparative study between the in vitro anti-inflammatory properties of a methanolic extract of *Tosochrysis lutea* (*T. lutea*) F&M-M36, a marine microalga belonging to the Haptophyta, and the single xanthophyll fucoxanthin (FX), on RAW 264.7 macrophages stimulated by lipopolysaccharide (LPS) [7]. To date, the anti-inflammatory and antioxidant effects of *T. lutea* have been mostly attributed to FX, one of its major constituents. However, *T. lutea* is also a source of phenolic compounds with a large spectrum of biological activities, including antioxidant, antiaging and anti-inflammatory effects, so positive pharmacodynamic synergies among the various components, acting on different targets, cannot be ruled out. In this study, the authors showed that the methanolic extract of *T. lutea* F&M-M36 exerts promising anti-inflammatory activity against COX-2/PGE_2_ and NLRP3/mir-223, even more pronounced than that of FX alone, which could be attributable to the known anti-inflammatory effects of the simple phenolic compounds found in the extract. These phenolic compounds could synergize with FX, and *T. lutea* F&M-M36 could serve as a source of anti-inflammatory compounds to be further evaluated in in vivo models of inflammation.

Among naturally occurring biomolecules, polysaccharides derived from algae and marine plants have received increasing attention among researchers. Indeed, marine polysaccharides possess numerous health benefits as well as raw materials for the pharmaceutical, nutrition and cosmetic industries. A wide variety of natural products with seaweed polysaccharides, with recognized antioxidant effects, are gradually entering people’s line of sight.

In this context, Yang et al. (2021) revealed to the scientific community the ameliorative effects of polysaccharide extract of *Ulva lactuca* (UPE), a type of green alga from coastal areas of China, on d-galactose (d-gal)-induced oxidative stress renal damage [8]. The intragastric administration of UPE in mice subjected to subcutaneous injection decreases serum creatinine, blood urea nitrogen and serum cystatin C levels; increases the glomerular filtration rate; enhances antioxidant enzyme activities; and reduces biomacromolecule damage caused by oxidative damage. It also significantly reduces the levels of inflammatory cytokines caused by oxidative stress. In addition, UPE administration could prevent the d-gal-induced apoptosis of renal tubule cells. This study highlights the protective effects of UPE on renal injury caused by oxidative stress, providing a new theoretical basis for the treatment of oxidative damage diseases.

Overall, marine-derived bioproducts are attracting incredible interest and appear to be a revolutionary therapy for various inflammation-related diseases, due to their beneficial health properties, which are attributed to the presence of characteristic biologically active functional constituents. In this context, research is ongoing worldwide, and researchers have redirected or reclaimed their interests in exploiting natural biological entities/resources, such as the algal biome. In this regard, Bilal et al. (2021) compiled a review that presents some natural biological compounds derived from the algal biome for the efficient management of inflammatory bowel diseases (IBDs), a chronic inflammation of the gastrointestinal tract [9]. A large number of marine bioproducts have been purified and identified from marine sources and have shown antioxidant properties and anti-inflammatory effects. However, the need for further research efforts has emerged to inspect the bioavailability and efficiency of marine bioproducts in human and animal models in order to prevent and manage IBDs.

Marine biotechnology aims to discover bioactive molecules from marine organisms to reveal their functions and actions, but also to understand the genetics, biochemistry, physiology and ecology of the marine organism. In light of this, Roncalli et al. (2021) provided the first piece of evidence of the presence of the OvoA gene, a key player in the ovotiol biosynthetic pathway, in arthropods [10]. Ovotiol is one of the most potent antioxidants that acts in marine organisms as a defense against oxidative stress during development and in response to environmental cues. Through a transcriptomics analysis, a single OvoA gene was found in marine arthropods including copepods, decapods and amphipods. Additionally, in particular, changes in OvoA gene expression through development and under stress conditions suggest that ovotiol may play a role as a defensive compound in *C. finmarchicus*, thus proposing this gene as a novel biomarker of stress in holozooplanktonic species. This discovery sheds light on copepods as marine organisms capable of producing bioactive compounds, opening further possibilities both for drug discovery and in the field of marine biotechnology.

In conclusion, as summarized in Figure 1, the studies collected in this Special Issue confirm that deep-sea organisms represent an extraordinary source of bioactive molecules with antioxidant and anti-inflammatory activity that can direct research to the design of new drugs.

## Figures and Tables

**Figure 1:**
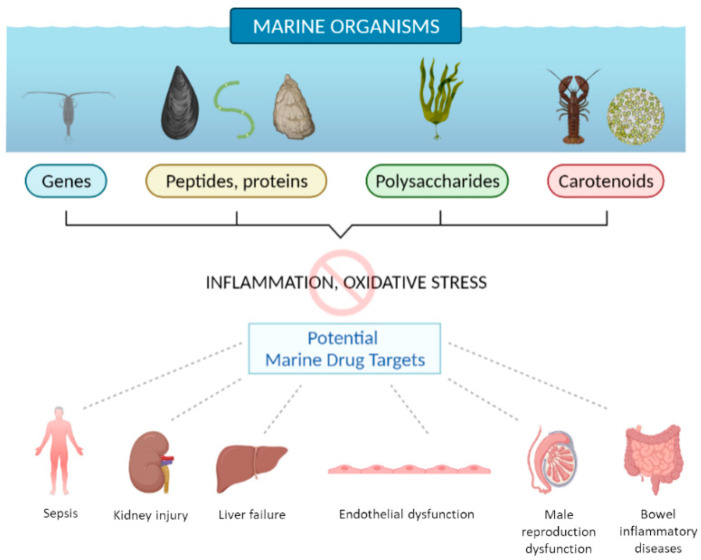
Schematic representation of the beneficial human health properties of biomolecules from marine organisms covered in the Special Issue “Marine Anti-inflammatory and Antioxidant Agents 2021”, created with https://biorender.com/ (accessed on 5 January 2022).
